# Editorial: Listening in action: Attention, emotions and cognition in the auditory system

**DOI:** 10.3389/fnins.2022.1007095

**Published:** 2022-08-22

**Authors:** Diego Elgueda, Yaneri A. Ayala, Paul H. Delano

**Affiliations:** ^1^Departamento de Patología Animal, Facultad de Ciencias Veterinarias y Pecuarias, Universidad de Chile, Santiago, Chile; ^2^Instituto de Neurobiología, Universidad Nacional Autónoma de México Campus Juriquilla, Querétaro, Mexico; ^3^Departamento de Neurociencias, Facultad de Medicina, Universidad de Chile, Santiago, Chile; ^4^Servicio de Otorrinolaringología, Hospital Clínico de la Universidad de Chile, Santiago, Chile; ^5^Centro Avanzado de Ingeniería Eléctrica y Electrónica, AC3E, Universidad Técnica Federico Santa María, Valparaíso, Chile

**Keywords:** bottom-up and top-down control, auditory attention, medial geniculate body (MGB), olivocochlear efferent, auditory system, pupil dilation response

Humans, as well as animals, can perceive pressure changes in their environment as sounds, and relate these sensations to their current behavioral goals, memories and the environmental context in which they are immersed. This allows them to communicate and to adapt to their surroundings by modifying their behavior to enhance their survival and reproduction chances. This is allowed by the ability of the auditory system, and the brain as a whole, to form meaningful auditory perceptual objects integrating incoming acoustical and top-down non-acoustical information (Bizley and Cohen, 2013). The structural substrate for this integrative process lies in the massive interconnection of the auditory system with areas of the brain related to executive, emotional and association cognitive functions, as well as those involved in the perception of other sensory modalities (Hackett, 2011; Elgueda and Delano, 2020).

Despite active research in this subject, our knowledge on how auditory perception is actively shaped by cognition, behavior, emotions and movement is still incomplete. The aim of this Research Topic was to incorporate original research exploring the role of cognition in shaping auditory perception, from the cellular to the systems perspective.

One of the most fascinating functions of the auditory system is its ability to extract and represent attended sounds from among competing sources. In this regard, it has been shown in human subjects that the auditory cortex can enhance the representation of attended speech relative to unattended sound sources (Mesgarani and Chang, 2012). This allows listeners to focus on speech in crowded environments. Unfortunately, current assistive hearing devices cannot yet select which sound sources are being attended, which makes it challenging for hearing-impaired listeners using them to extract speech from interfering noise. This has motivated, in recent years, the research on neuro-steered hearing aid devices that monitor neural activity in the subject and compare it with sound sources in the environment to determine the attended sound source (Geirnaert et al., 2021). This demands for robust real-time Auditory Attention Decoding (AAD) algorithms capable of detecting the attended stream. In this Research Topic, Cai et al. propose a novel AAD model based on a recent architecture in machine learning, Cross-Modal Attention (CMA), which dynamically captures the correlations between neural and audio signals—resembling top-down and bottom-up inputs. This new model was validated using a publicly-available database, showing that it can outperform conventional linear AAD algorithms as well as existing non-linear approaches, achieving high-accuracy classification even for short time windows and in real-world reverberant conditions.

The extensive interconnection of the auditory system with non-auditory cortical and subcortical structures allows auditory information to be preattentively evaluated for novelty. This function is critical for survival, as it permits individuals to detect unexpected stimuli in order to determine its behavioral relevance and react with appropriate adaptive responses. Unexpected sounds, as well as cognitive load, arousal and attention have been reported to cause changes in pupil dilation in humans and non-human primates, which has motivated the measurement of pupil dilation as a non-invasive proxy for cognitive and emotional processing. Nonetheless, it's not yet clear whether the underlying neural bases for pupil dilation, as well as for processing unexpected stimuli, are comparable in both species. Selezneva et al. investigated this by comparing pupil dilation in humans and macaques involved in an auditory oddball paradigm, and found that both species present similarities, albeit monkeys showed shorter latencies and greater dilation amplitude than humans. In both species, it was found that pupil dilation responses consist of an early (likely associated to parasympathetic activity) and a later (likely sympathetic) component, suggesting that macaques are a suitable model to study the biological underpinnings of pupil dilation in humans.

Subcortically, the Medial Geniculate Body (MGB) in the thalamus is a critical hub for auditory pathways; all ascending information from auditory subcortical stages must pass through the thalamus before being relayed to the cortex, while also receiving many excitatory and inhibitory descending inputs from the cortex and other brain regions (Hackett, 2011). This places the MGB at a pivotal point for the integration of bottom-up and top-down auditory information. However, it is not yet clear how ascending and descending information are integrated at the neuronal level. Fujimoto et al. characterized the subcellular distribution of Kv4.2 potassium channels at synaptic level in the ventral division of the MGB (MGBv) of the mouse, since these channels may have an important role in shaping synaptic and spike waveforms while integrating the different temporal resolutions of inputs coming from the inferior colliculus (fast, high resolution responses) and auditory cortex (lower temporal resolution). They described that most MGBv neurons display expression of Kv2.4 channels at both the dendritic and somatic levels, and that the Kv2.4-rich dendritic domains receive preferentially ascending excitatory and inhibitory inputs coming from the inferior colliculus and not from top-down sources, implying that these voltage-gated potassium channels might be important in shaping the responses elicited by ascending information.

Finally, Alvarez-Munoz et al. reviewed the auditory efferent network, focusing on corticofugal and brainstem circuits involved in audition and cognition. The authors emphasize on behavioral studies performed in knock-out mice for the α9-nicotinic receptor subunit, which lacks cholinergic transmission between medial olivocochlear neurons and outer hair cells. Importantly, the lack of efferent transmission does not alter hearing thresholds, but more complex auditory functions, such as frequency selectivity or cognitive functions, such as filtering of auditory responses during visual attention are affected.

This Research Topic features original research that adds to our accumulated knowledge on how higher cognitive functions can influence hearing, and provides new insights that can enable future studies to better comprehend how the auditory perception can be shaped by our experience, emotions and cognition, while also helping in the development of new technologies for assisted hearing devices.

## Author contributions

All authors listed have made a substantial, direct, and intellectual contribution to the work and approved it for publication.

## Funding

DE was funded by FONDECYT 11190278 and 1220607 ANID. PD was funded by FONDECYT 1220607, ANID Fondo basal FB0008, and Proyecto Milenio ICN09_015.

## Conflict of interest

The authors declare that the research was conducted in the absence of any commercial or financial relationships that could be construed as a potential conflict of interest.

## Publisher's note

All claims expressed in this article are solely those of the authors and do not necessarily represent those of their affiliated organizations, or those of the publisher, the editors and the reviewers. Any product that may be evaluated in this article, or claim that may be made by its manufacturer, is not guaranteed or endorsed by the publisher.
